# Adolescent Obesity and Social Networks

**Published:** 2009-06-15

**Authors:** Laura M. Koehly, Aunchalee Loscalzo

**Affiliations:** National Institutes of Health; National Human Genome Research Institute, Bethesda, Maryland

## Abstract

The prevalence of overweight among children worldwide is growing at an alarming rate. Social relationships may contribute to the development of obesity through the interaction of biological, behavioral, and environmental factors. Although there is evidence that early environment influences the expression of obesity, very little research elucidates the social context of obesity among children or adolescents. Social network approaches can contribute to research on the role of social environments in overweight and obesity and strengthen interventions to prevent disease and promote health. By capitalizing on the structure of the network system, a targeted intervention that uses social relationships in families, schools, neighborhoods, and communities may be successful in encouraging healthful behaviors among children and their families.

## Introduction

The prevalence of overweight among children has tripled in the last 40 years. Although recent data suggest that childhood overweight rates have begun to plateau, 32% of youth aged 2 to 19 years are overweight or at risk of becoming overweight (1,2). Furthermore, childhood overweight contributes to type 2 diabetes, adult obesity, and heart disease, along with impaired self-esteem and depression (3).

Adolescent overweight is largely a product of familial obesity risk (4), but environmental influences can augment the expression of overweight in children with a family history of obesity, continuing into adulthood. A social network approach to research and intervention design accounts for social contexts such as family, schools, neighborhoods, or communities, revealing how people are interconnected and influence one another. A social network approach is a relational perspective that frames research involving individuals and their families and communities, in addition to the methodologic tools that are used in social network analysis. We discuss the use of a social network approach in interventions for adolescent overweight by considering 1) recent developments in the science of obesity genetics, 2) the importance of social context, 3) communal coping as a mechanism for behavior change within social networks, and 4) specific recommendations for using social networks to prevent overweight.

## Obesity and Family History

A recent study estimates that more than 70% of adiposity in 10-year-olds is due to genetic factors, and approximately 20% is due to socioenvironmental contributions (4). Genome-wide association studies have located common genetic variants associated with fat mass, weight, and susceptibility to obesity. Several genes isolated through these studies, including *FTO* (5) and *MC4R* (6), may eventually help scientists to explain the global scale of the obesity epidemic and the biological mechanism for the heritability of obesity in families.

Other research has identified factors associated with the behavioral transmission of obesity risk from parents to their children (7). Eating disinhibition, susceptibility to hunger, and eating in the absence of hunger all appear to be biologically heritable traits. Thus, a child's family health history, along with shared behaviors and familial environments, must be considered in efforts to prevent and treat obesity (8).

## Early Social Environments and Overweight

Excessive caloric intake and a lack of physical activity are 2 major environmental causes of adolescent overweight. Both structural and behavioral environments in which adolescent social networks operate are inextricably linked to their eating behaviors and physical activity levels.

Early childhood feeding practices are usually established in the home and often translate into eating patterns during adolescence. Variations in food preferences and portions among preschool children are associated with the extent to which parents introduce new foods and encourage healthful eating habits (9). Moreover, maternal feeding practices appear to influence the dietary patterns of girls, suggesting that the relational significance of parental influence on their children may be sex-specific (10).

Likewise, early childhood activity levels translate into similar patterns of physical activity during adulthood (11). Physical activity among adolescents is a social behavior, which is partly dependent on neighborhoods and recreational spaces. Built environments can limit or facilitate levels of adolescent physical activity. Playgrounds that are accessible via sidewalks and safe intersections have been associated with higher levels of physical activity among youth (12).

## Adolescent Overweight and Social Networks

Mutual friendship ties, not merely biological family or relationships found within the household, can contribute to an adult's risk of obesity (13), but little is known about whether the social mechanisms associated with weight gain in adults pertain to adolescents. Studies of adolescent social networks have identified the extent to which clique formation, the tendency for people to form social ties with others who are similar (14), are associated with weight status and physical activity. One study found that adolescent friendships tended to cluster on the basis of weight status (15). The boys who were friends engaged in similar levels of physical activity; however, this finding was not noted within girl friendship networks (16). Another study found similarities in the consumption of sweet foods and fast foods and types of physical activities among male friends, and female friends were similar in the time spent on computer-based leisure activities (17).

The mechanisms of social influence on adolescent overweight vary, but all depend on social interaction. Parents can serve as role models, especially for younger children whose health behaviors are completely influenced by their parents' habits (18), and older children may look to their friends, teachers, and community leaders as role models for their own health behaviors (19). Indirect processes can occur through cultural or group norms and attitudes. For example, adolescents' attitudes about body image can be influenced by social and cultural norms (20).

## Communal Coping

A social network approach fits within a socioecological model for obesity interventions, because social networks form and operate within the social contexts that influence health behaviors and behavior change (21). Capitalizing on these interpersonal relationships may enhance the effectiveness of health promotion interventions (22).

Communal coping is a process in which interpersonal relationships are the conduit to behavior change among multiple members within a particular social network, such as families (23). Its use in obesity prevention is novel, because it prioritizes relational over individual processes. From a communal coping perspective, individuals define themselves in terms of their interconnectedness and relationships with their family, friends, neighbors, and community. Thus, when faced with a shared health problem, a cooperative approach to address the problem that involves family and friends may be particularly effective (23).

**Figure 1:**
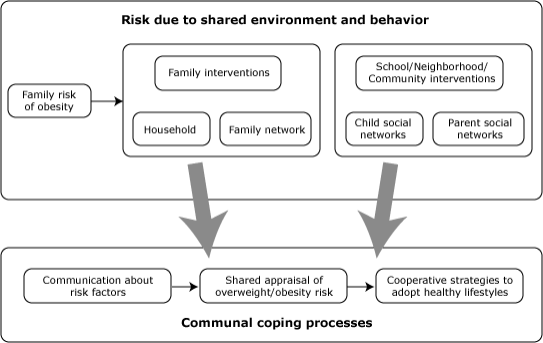
The communal coping framework. This illustration shows the pathways through which increased risk due to shared genes, environment, and behavior may lead to the process of communal coping.

Health interventions that use communal coping can target 3 interpersonal pathways (Figure 1): 1) communication about a health problem, such as shared risk factors, 2) shared appraisals of the problem, and 3) development of cooperative strategies to reduce negative impact (23). Interventions can focus on educating family members about collective risk due to shared family history, environment, and behaviors, and promoting increased communication about family risk of overweight and associated diseases. Similar efforts can motivate communication about shared risk factors among friends in neighborhoods and communities, leading to shared appraisals among those who are socially connected. The success of communal coping depends on cooperative support mechanisms. Support can be directed at emotion-focused coping to address, for example, low self-esteem or psychological impacts of stigma associated with overweight and obesity. Cooperative support also can be geared toward problem-focused coping by addressing dietary behavior and physical activity.

## Using Social Network Approaches to Strengthen Obesity Prevention

Obesity prevention must account for the complexity of overweight, including a child's familial risk of obesity and social relationships. Most previous interventions have focused on a single social sphere, such as household or school. Furthermore, family-oriented interventions often engage an affected child and a single caregiver, rather than considering the complex social environment that might surround children and their families. An intervention that focuses on the family system will have limited success without consideration of the social influences on both parents' and children's behaviors outside of the family context. Similarly, a school-based intervention that does not consider the familial social environment or interpersonal influences within the neighborhood or community settings would also be limited.

**Figure 2:**
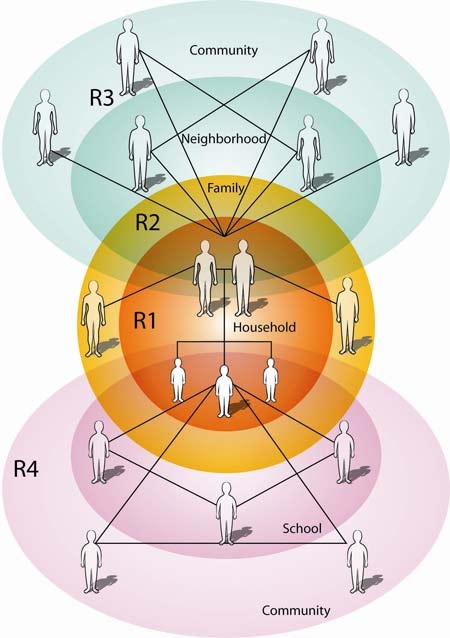
Recommendations ("R1" through "R4") to prevent and control adolescent overweight. This illustration shows how social networks of children and parents interconnect with other social contexts that are important to obesity prevention in adolescents.

Thus, we recommend that interventions focus on 3 settings simultaneously (Figure 2, Table): R1 and R2) the household and the child's family outside of the household; R3) the neighborhood and community, to engage the parents' social network and social influences on the child outside of the school setting; and R4) the school, to engage the child's social network.

### Recommendation 1: Intervene with the family system, rather than with the individual.

Primary prevention efforts may be more effective if they focus on the home environment. To date, household-based interventions have been largely focused on treatment of childhood overweight but not primary prevention. A detailed family history capturing the constellation of family members who are overweight, and associated diseases, can identify at-risk families for primary prevention efforts. Adolescents can be engaged in the process of gathering family health history of chronic illness and associated risk factors such as overweight, which will provide an opportunity for families to communicate about their shared risks (24).

Interventions based on the communal coping model could initially focus on facilitating communication among household members and educating them about their shared risk of disease. This process should engage multiple family members and not be limited to an at-risk child and the primary caregiver. The key to activating communal coping is to develop common appraisals of a shared health threat among group members. In the case of a family-based intervention, a risk assessment based on family history might motivate the perception of risk of overweight as a household-level problem, warranting a household-level solution. One model for developing risk assessments based on family risk is Family Healthware. This software, developed by the Centers for Disease Control and Prevention, produces an evaluation of an individual's risk of disease based on their family health history and recommends health behaviors that may reduce their risk.

### Recommendation 2: Tailor family-based interventions to the structure of the family.

Because families are complex social systems, family-based interventions must be flexible enough to adapt to the unique needs of individual families. Social network approaches can be used to gather information on the existing configuration of relationships within families, including family members outside of the household, the composition of the family, the functional significance of family ties, and the way social influence functions in each family. This information can be used to determine the key people within the family who might be able to exert a strong enough influence to change behavior. These optimally positioned family members may receive training and education and be engaged as "family leaders" who encourage cooperative strategies to increase physical activity, prepare healthy meals, and provide moral support that may help to sustain long-term behavior change within the family.

Cultural and sociodemographic factors are associated with the way families are organized, the social significance of food, food preferences and eating behaviors, and the way children are socialized. A formative assessment can elucidate shared beliefs and behaviors of families that may not be apparent through structural analysis. Such knowledge can lead to the design of culturally appropriate intervention materials, which can then be implemented according to unique family characteristics.

### Recommendation 3: Design support mechanisms for parents and adult family members on the basis of their social ties within the community.

Preventing childhood overweight is likely to have the most sustainability when it is implemented early in the immediate family environments of young children and continues through interventions in schools, neighborhoods, and other community settings. Young children often model the health behaviors of parents and other adults in their lives (18,19). To be positive role models for the children in their homes, adult family members may need to change their own lifestyles.

At the same time, the health of adult family members depends somewhat on their social ties (13). Social ties and the structure of these ties can affect behavior and health through social influence, social support, access to resources, and access to information. Social psychological theories suggest that a person's friends are likely to share similar lifestyle behaviors, such as diet and levels of physical activity, and thus be at similar risk for overweight.

One way to support obesity interventions among influential adults in adolescents' lives is to consider the social ties that influence adult eating and physical activity behaviors. For example, using a social network approach, cohesive subgroups of friends within neighborhoods and communities could be identified for health promotion. A central person within the group can be identified on the basis of the network's structure and trained to act as the liaison between the friendship network and the intervention team. The peer leader can create opportunities for discussing lifestyle risk factors, provide educational materials developed by the intervention team, and organize group activities aimed at promoting healthful lifestyles among friends. By using the naturally occurring structure of a group of friends, the designated leader will have credibility within the group and be more effective in relaying helpful information on diet and physical activity.

Neighborhood or community health promotion activities can be designed and targeted to these friendship networks. For example, neighborhood "house parties" may provide opportunities for friends to meet monthly, preassemble healthy meals, and discuss educational materials with tips on how to provide a healthy diet to their family. A similar approach can be used to increase physical activity, for example, a coordinated effort within the friendship network to exercise together several times a week. Targeting intervention activities to the natural groupings of friends capitalizes on the social influence processes inherent within friendship networks as well as the continued provision of social support and encouragement of healthful lifestyles.

### Recommendation 4: Use peer networks to encourage increased physical activity.

Because adolescents seem to cluster according to physical activity levels (15,17), network-based interventions may be particularly effective in developing coordinated physical activity efforts among adolescent friends. The most popular of these interventions encourages change in friendship networks through a peer leader, a central influential person, or opinion leader selected on the basis of the structure of social ties among the children within a classroom or community organization. This approach is easy to implement, has been effective in smoking prevention interventions (25), and has the potential to increase physical activity among adolescents.

Overweight adolescents are often socially isolated, which in turn may lead to "emotional eating" (3). Social network interventions might focus specifically on helping isolated overweight adolescents form new social ties that have health benefits. Classrooms and community settings are ideal for such activities. A buddy system between people who were previously unconnected has been successful in reducing social isolation; this peer-teaching intervention involved older-younger schoolchildren pairs (26).

Team-based physical activity has been effective for weight reduction and lifestyle change when at-risk and overweight youth were members of organized sports teams (27). Motivating overweight youth to participate in these team-based activities may require special support, such as school-based policies and community programs designed specifically to meet the needs of children who are overweight. One social network approach to encouraging participation in organized sports could involve assigning team membership based on naturally occurring friendships and cliques among overweight youth. This strategy would simultaneously increase physical activity levels, encourage positive peer influences on weight reduction, and reduce social isolation (3).

## Conclusion

According to Barabasi, "Growing interest in interconnectedness has brought into focus an often ignored issue: networks pervade all aspects of human health" (28). Network perspectives will continue to advance the study of childhood and adolescent overweight. We suggest a new and stronger focus on the potential to garner interpersonal processes to address the obesity problem. Consideration of family and social networks may contribute to sustainable behavior change and improve the effectiveness of prevention and treatment interventions. Although challenging, curbing the obesity epidemic will undoubtedly depend on the coordinated efforts of many agencies and institutions to support culturally sensitive programs that consider both family and peer interactions.

## Figures and Tables

**Table.** Network-Based Interventions to Prevent and Control Youth Overweight

**Focal Network^a^**	**Network/Structural Components**	**Intervention Component(s)**	**Communal Coping Mechanism**	**Desired Outcome**
Family system (R1)	Construct family pedigree and family health history Enumerate household network Characterize family network structure beyond the household	Provide information about genetics and heredity of obesity Provide family risk assessment based on family history and shared risk factors and help with interpretation	Family communication Shared appraisals of risk	Increased communication about obesity risk within the family
Family system (R2)	Identify influential members Define family roles in meal planning and preparation Perform formative assessment of physical activity level and diet for each network member	Tailor educational materials to the family roles within the network Identify activities and strategies to adopt healthy lifestyles tailored to meet varying needs of family members	Cooperative strategies to address behavioral risk factors	Increased engagement in behaviors to reduce risk of obesity Increased encouragement and social support among family members
Adult peer networks (neighborhood/ community; R3)	Characterize friendship networks within neighborhood and/or community Identify peer leaders within friendship clusters Identify household members involved in meal preparation and planning	Train peer leaders within friendship networks as lay health advisors Organize physical activity among members of adult friendship networks Provide neighborhood or community-based health seminars aimed at informing friendship networks about obesity-related risk factors and health concerns Introduce neighborhood meal planning and preparation activities	Shared appraisals of risk Cooperative strategies to promote physical activity Cooperative strategies to promote healthful eating	Increased encouragement and social support among friends Increased physical activity Increased consumption of healthy foods
Child peer networks (school/ neighborhood/ community; R4)	Characterize friendship networks within schools, neighborhood, and/or community Identify child's preferences for physical activity Identify peer leaders in friendship networks to promote physical activity Identify isolated persons and integrate into peer networks	Organize team sports defined by activity preferences Define team membership by friendship clusters Define team leaders by structure of friendship networks Organize peer teaching, co-engagement in physical activity, and support	Cooperative strategies to promote physical activity Cooperative strategies to promote physical activity, healthful eating, and social support	Increased physical activity Reduced social isolation Increased participation in activities to reduce disease risk Changed social norms regarding healthy lifestyles

a Focal networks are defined based on our recommendations (R1 through R4) to R1) intervene with the family system rather than the individual, R2) tailor family-based interventions to the structure of the family, R3) design support mechanisms for parents and adult family members on the basis of their social ties within the community, and R4) use peer networks to encourage increased physical activity.
